# Comprehending Alzheimer’s Patient and Caregiver Needs

**DOI:** 10.1002/alz.091451

**Published:** 2025-01-09

**Authors:** Johanne Stümpel, Marlena van Munster, Annika Dörrhöfer, Björn Schmitz‐Luhn

**Affiliations:** ^1^ Center for Life Ethics, Bonn University, Bonn Germany; ^2^ Philipps University Marburg, Marburg Germany; ^3^ Bonn University, Bonn Germany; ^4^ Center for Life Ethics, University of Bonn, Bonn Germany

## Abstract

**Background:**

Projected Alzheimer’s disease (AD) prevalence is expected to fourfold by 2050. With the escalating numbers of individuals affected by neurodegenerative diseases, including Parkinson’s disease (PD) and AD, associated challenges encompass increased burdens on individuals and families, societal and political implications and economic impacts. Integrating patient and caregiver perspectives is essential to creating comprehensive, patient‐centered care models that promote well‐being and resilience in the face of degenerative neurological diseases such as AD. This approach aims to contribute valuable insights into designing patient‐centered care interventions that can address common concerns across diverse neurodegenerative conditions.

**Methods:**

Ten interviews and co‐design workshops were conducted with PD‐diagnosed individuals and their caregivers to explore future care perspectives. Participants selected cards from three domains (‘Resources,’ ‘Technologies,’ and ‘Interactions’) to envision their care. Most frequently named items were cross‐referenced with existing AD literature. Thematic analysis identified recurring patterns, and rigorous coding ensured a comprehensive exploration of various viewpoints. Synthesizing findings and extracting key themes, we contextualized results through an exploratory literature review. The integration of diverse data sources provided a robust foundation for understanding future neurodegenerative disease care needs.

**Results:**

We identified needs and challenges throughout the disease trajectory, emphasizing emotional burden and daily life disruption. Recognizing similarities across neurodegenerative diseases, PD experiences may well be transferable to individuals with AD. However, existing AD literature proved inconclusive regarding patients’ and caregivers’ perspectives on future holistic care approaches. Our findings underscored interdependence of patient and caregiver experiences, highlighting the need for holistic support, also in AD. These address providers, administrators, and policy makers and call for tailoring technological advances, resource allocation, and strategies to the unique needs of each stakeholder group.

**Conclusion:**

The findings underscore the need for tailored interventions and support services addressing the unique needs of individuals with neurodegenerative diseases and their caregivers. Given the increasing global prevalence of AD, understanding and meeting these needs are critical to improving the quality of life for affected persons. Moving forward, these findings contribute valuable insights for the development of holistic care models, fostering a supportive healthcare environment for those affected by neurodegenerative diseases.

